# An empirical assessment of whether urban green ecological networks have the capacity to store higher levels of carbon

**DOI:** 10.1038/s41598-024-52650-y

**Published:** 2024-02-07

**Authors:** Yunshan Wan, Yilei Wang, Ming Gao, Lin Jin

**Affiliations:** 1 School of Architecture, China Architecture Design & Research Group, Beijing, China; 2 Department of Landscape Architecture, Huazhong Agricultural University, Wuhan, China; 3 School of Architecture, Harbin Institute of Technology, Harbin, China; 4 Key Laboratory of Cold Region Urban and Rural Human Settlement Environment Science and Technology, Ministry of Industry and Information Technology, Harbin, China; 5 Interdisciplinary Program in Landscape Architecture, Seoul National University, Seoul, Republic of Korea; 6 Integrated Major in Smart City Global Convergence, Seoul National University, Seoul, Republic of Korea

**Keywords:** Ecology, Environmental sciences

## Abstract

Carbon–neutral growth is a crucial long-term climatic aim in the context of global warming. This paper introduces complex network theory and explores its potential application to achieve this goal. Specifically, we investigate the spatial and temporal distribution of nodes and sources in the ecological network, and examine whether a relationship exists between the topological index of network nodes and the landscape pattern index of ecological source areas. We also determine the contribution of nodes to the carbon stock of the entire network by exploring the correlation between the carbon stock of nodes and sources to develop an optimization strategy based on the synergistic effect of node-source carbon enhancement. Finally, we test the effect of network optimization through robustness. Our results show that: (1) The correlation topological feature index analysis reveals that the degree distribution of the node network's topological characteristics becomes dispersed and modular, exhibiting the characteristics of small-world networks according to a large clustering coefficient. The heterogeneity and extent of ecological source landscapes have increased by modularity index but remain distributed and locally fragmented; (2) According to correlation analysis, by enhancing the eccentricity of the node topology, the patch cohesion index (COHESION) of the ecological source site can maximize the contribution of the node to the enhancement of the carbon stock benefits of the source site; (3) According to the tests on the robustness of nodes and edges and the robustness of network links, network stability is improved and carbon sink capacity is enhanced. Simultaneously, the restoration and rejuvenation of ecological space through national ecological construction projects can effectively improve the carbon sink within the organized region, contributing to the carbon neutrality aim. This research gives scientific and quantifiable references for potential ecological construction projects for sustainable cities and the optimization of urban ecological space structure.

## Introduction

Under the backdrop of swift urbanization, the ecological networks within green spaces are confronted with environmental issues, including weak resilience to disturbance, limited capacity to provide ecosystem services and a decline in biodiversity. This situation reduces the capacity of green spaces to sequester carbon and prevents the regional ecological network from reaching its maximum potential as a carbon sink. As a proactive supporter of green and low-carbon development, China recognizes sustainable green development models to confront the negative effects of global climate change. Constructing a rationally designed green space ecological network is crucial for integrating patterns, processes, and functions and has significant potential to offset the carbon footprint of cities. Quantifying the carbon metabolic efficiency of urban green spaces, and understanding their compositional relationships and mechanisms of action, is a practical approach to achieving spatial optimization^1,2^.

Numerous scholars have emphasized the significance of green ecological networks in enhancing carbon sinks in green space systems, which are composed of core areas and ecological connections^3–5^. This provides an important spatial foundation for promoting the ecological processes of material and energy cycles in city clusters^6,7^. In addition, integrating carbon metabolism systems with ecological network analysis has become a system-oriented approach. It supplies a macro-scale perspective to assist low-carbon development and coordinate urban carbon metabolism, including the impact of indirect and critical pathways^5,8,9^. From a network perspective, it is possible to identify and quantify the characteristics of spatial and temporal carbon flow exchange resulting from land use changes. This approach helps identify the processes associated with carbon stock increases and decreases, enabling a better understanding of how to balance the carbon budget and promote sustainable urban development^10–12^. Researchers are able to investigate how different land use patterns impact urban carbon neutrality during urbanization and how they can be optimally adjusted to reduce or eliminate carbon metabolism disorders in cities. By exploring such questions, researchers can better understand the mechanisms underlying urban carbon balance and propose sustainable solutions for harmonizing the carbon balance in urban areas^4,13,14^.

Multiple studies present that changes in the structure of green space networks can enhance sink capacity by influencing ecosystem resilience, robustness, energy flow, cycling, and overall function. These changes can impact an area's regional carbon sequestration capacity by affecting its overall function and stability^6,7,15–17^. For instance, vegetation is widely considered the best carrier for carbon capture and storage, but blindly planting trees without considering the structural layout of the green space network can lead to neglecting other factors., such as landscape, climate, and hydrology, which also impact tree growth. This makes it challenging to achieve simultaneous increases in network connectivity, ecosystem services and carbon sequestration services. Therefore, in the future, the planning and layout of green space systems in China should prioritize the transformation from quantity to quality within a limited space, explore the optimal ecological spatial pattern and maximize the effect of carbon sink enhancement. Furthermore, improving the level of carbon sinks in green ecological networks involves combining ecological networks with complex networks. Complex networks provide a means to extract and evaluate ecosystems and landscape spaces within the study area. When applied to a complex network, topological indicators could be utilized to examine the relationships between network parts and offer practical optimization options based on indicator values^18–20^. By integrating ecological networks with complex network theory, ecological corridors and ecological sources could be represented in ecological spatial networks as edges and nodes, simplifying them and making them more systematic. Simultaneously, topological indicators derived from complex network theory could be utilized to analyze the relationships between network components, reflecting the topological characteristics of the ecosystem and ultimately proposing particular optimization instructions according to the topological values results. Complex network theory provides powerful tools for quantifying the spatial relationships and dynamics of ground objects. By analyzing the spatial distribution and composition of ground objects, as well as their topological relationships, topological indicators derived from complex network theory offer an effective means of capturing and measuring these relationships and changes between ground objects in terms of their spatial location.

Concerning the application of the theory, many scientists have employed the topological properties of complex network theory to analyze the structure of ecological networks and verify that the state of each element is accurate in the networks. This allows scientists to determine the areas that need optimization using the above topological indicators of a complex network^6,21–23^. Additionally, several strategic approaches have been proposed to improve the connectivity of nodes, edges, and networks. These approaches aim to improve functional–structural synergy for an enhanced carbon sink arrangement. For instance, scientists suggest connecting fragmented ecological source sites and adding corridors between lower-grade sources by placing stepping stones at corridor breakpoints. By doing so, scientists want to create an ecological network area where ecological functions and carbon sinks can work together more effectively^24–27^. Other scientists have attempted to establish the connection between ecological spatial relationship networks. Carbon sinks and carbon emission capacity, proposing further bases for the optimization of the green space system and decarbonization measures for the overall urban design^28–30^.

In general, systematic studies linking carbon sink issues to ecological networks are still in their infancy, with many scholars currently only simulating studies that focus on ecological spatial networks for a specific year, with few cases examining ecological and carbon sink issues at the city scale. Current studies of carbon storage at the spatial level focus more on the variability of multiple land classes but rarely focus on the spatial quantification of the quality of extraction itself, thus providing a basis for optimizing the spatial pattern of organizing the carbon storage capacity of each of the ecological elements^31–33^. Studies on the network level are more likely to focus on a wider range of ecosystem service types^32,34,35^, with a systematic approach to spatial quantification providing a scientific and feasible design basis for a more systemic spatial design purpose^36^, thus providing a broader perspective on the pressing issue of 'carbon neutrality' spatial implementation. By combining carbon sinks and ecological networks, carbon storage network research can establish a systematic relationship between land cover type changes and the spatial layout of carbon sinks. Also, introducing an ecological network approach to the study of carbon storage can help us build a carbon storage network to optimize the topological indicators of nodes and edge enhancement strategies to drive the carbon storage capacity of the whole network. Namely, the topology of nodes can be optimized, with the edge enhancement strategy driving the carbon storage capacity of the network. Currently, some scholars have made specific suggestions on the study of carbon sink networks, such as the construction of a spatial optimization scheme for mining cities to enhance ecological services and sink functions simultaneously^37^ or the clear correlation between topological characteristics and carbon nodes^25^. However, none have addressed the important components of the carbon sink network, including the specific correlation between source sites and nodes. This is where this current study differs from previous research. This is the three-way relationship respectively that needs to be considered between the topological network of nodes and the pattern of the landscape of sources, the carbon sink capacity of nodes and sources, and the screening of optimization points based on the capacity characteristics of node-source site carbon sink buffers, which will then allow us to give spatial optimization recommendations based on the aforementioned corresponding correlations.

Based on previous research and the knowledge gap, the following questions will be addressed by this study: (1) What features of the spatiotemporal variations in carbon sinks at nodes and source locations within the green ecological spatial pattern of Beijing-Tianjin-Hebei, as determined by ecological spatial network analysis? (2) What is the correlation between the spatial landscape structure index and network topology index, and how do they affect the effectiveness of carbon sinks? (3) What feasible optimization strategies can be proposed to enhance the synergistic effect of increasing carbon between nodes and source sites? This research seeks to develop a method for limiting the landscape structure and network topology indices in order to maximize the synergistic effect of nodes and source locations on carbon sequestration. Specifically, the study aims to optimize the carbon sink capacity of ecological source sites in the Beijing–Tianjin–Hebei city cluster by reducing the number of weak carbon sink nodes. It will also include recommendations to maximize the effect of carbon sinks at source sites.

## Materials and methods

**Figure 1:**
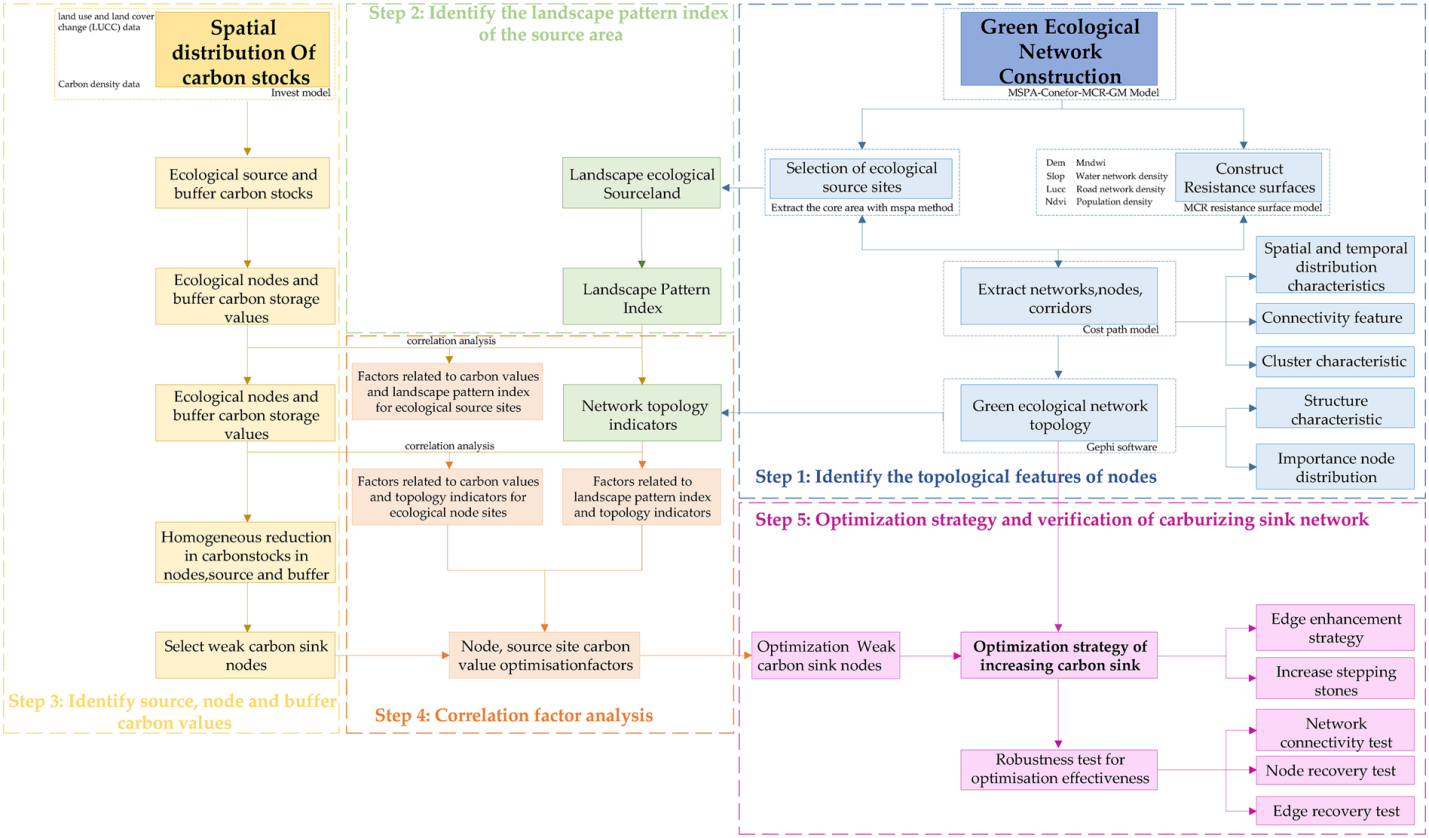
Technology roadmap.

The study is comprised of three parts. The objective of the first section is to ensure the feasibility of the ecological network by introducing the MSPA–Conefor–MCR–GM method (Morphological Spatial Pattern Analysis—Landscape Connectivity Analysis—Minimum Cumulative Resistance Model—Gravity Model). This method is used to construct a complete ecological network by integrating green ecological spatial elements and verifying important ecological nodes and corridors using the gravity model (Fig. S1, supplementary materials of urban green ecological networks model illustration). The Invest model is employed in the second part to calculate and evaluate the green ecological space elements and carbon storage value of important ecological nodes, patches, and their buffer zones. The third part investigates the connection between the topological properties of ecological nodes, pertinent markers of landscape patterns and carbon storage capacity. For nodes and corridors with weak carbon sinks, optimization techniques and strategies are suggested. The impacts of optimization are then confirmed by computing carbon sinks and conducting robustness tests on the network before and after optimization. The specific methods and steps for each part of the study are described in (Fig. 1).

### Study area

The Beijing-Tianjin-Hebei metropolitan area, situated in the central region of the Bohai Sea in the Northeast Asian region of China, is one of the most prosperous and largest metropolitan areas in the country, with a temperate monsoon climate and four distinct seasons. It lies between 36° 05′–42° 40′ latitude and 113° 27′–119° 50′ longitude east, with the topography of the entire region sloping from northwest to southeast at an average elevation of 1600–1800 m in the southwest and 1000–1200 m everywhere else. Due to policy changes and urban development, there have been significant industrial adjustments in Beijing, Tianjin and Hebei in recent years. The original industrial structure of the region was relatively rough and comprised of mainly manufacturing industry clusters with these industries contributing significantly to carbon emissions. The region's total carbon emissions account for more than 10% of the national share, making the region critical to achieving the carbon–neutral goal. Moreover, ecological sources in the region are gradually fragmenting, with some rivers and lakes drying up and breaking down, as well as recurring ecological problems, including sandstorms, haze, soil erosion, and water shortages. Therefore, there is an urgent need to improve the ecological quality of the Beijing–Tianjin–Hebei region in order to increase the number of carbon sinks and strengthen the regional ecosystem.

### Data sources

This study utilized the following multiple datasets from the years 2000, 2010 and 2020: (1) Beijing–Tianjin–Hebei elevation (DEM) and slope data were acquired from the Resource and Environment Science and Data Centre of the Chinese Academy of Sciences (https://www.resdc.cn/, accessed on 15 October 2022), with the study area data obtained via clipping in ArcGIS 10.8; (2) 30 m spatial resolution land-use data from the CLCD data of Yang Jie and Huang Xin’s team at Wuhan University with a spatial resolution of 30 × 30 km^38^ (https://doi.org/10.5281/zenodo.5816591, accessed on 15 October 2022); (3) 30 m NDVI and MNDWI data were calculated using the Landsat5 and Landsat8 datasets from the Google Earth engine data analysis platform (https://earthengine.google.com/, accessed on 15 October 2022); (4) Population density information was retrieved from WorldPop (https://www.worldpop.org/, accessed on 15 October 2022); (5) Road network and water network data were acquired from OpenStreetMap (https://www.openstreetmap.org/, accessed on 15 October 2022), with the road network density and water network obtained from ArcGIS 10.8 using kernel density analysis. We downloaded these data and then put them into ArcGIS 10.8 for visualization (Fig. S2).

### Methodology

#### Ecological cyberspace construction

##### MSPA method-based ecological source identification

Core landscape types in the ecological space were selected by utilizing the Morphological Spatial Pattern of the Landscape (MSPA) method, with the ecological source sites selected using connectivity analysis software (Conefor2.6). The grid image is precisely recognized and segmented into seven main categories of the landscape, with the ecological sources then identified using ArcGIS 10.8 to calculate the core patches and Conefor software used to calculate thresholds for selecting the potential connectivity index (PC) and patch significance index (dPC), with the following formula used to calculate the dPC.

1 $$PC = \left( {\mathop \sum \limits_{I = 1}^{n} \mathop \sum \limits_{j = 1}^{n} a_{i} \cdot a_{j} \cdot p_{ij}^{*} } \right)/A_{L}^{2}$$

2 $$dPC = \left( {PC - PC_{rem} } \right)/PC \times 100$$

$PC$ refers to the possible connectivity index, $a_{i}$ and $a_{j}$ are the areas of patches i and j, $A_{L}^{2}$ represents the overall area of the entire landscape, n refers to the overall number of patches in the landscape, $p_{ij}^{*}$ is the highest likelihood index for species migration between patch $i$ and patch $j$. $dPC$ can reflect the crucial patches, where the higher the dPC value, the more essential the patches are in the landscape's connectivity, and $PC_{rem}$ refers to the connectivity index of the remaining patches after deleting individual patches.

##### Extraction of ecological corridors according to the MCR-GM model

The minimum cumulative resistance model (MCR) was initially offered by Dutch ecologist Knaapen^39^ and used to calculate the minimal cost necessary for the movement of material flows from source to destination. The minimum cumulative resistance model (MCR) is constructed by combining eight ecological resistance factors in this study, including the digital elevation model (DEM), slope (SLOPE), normalized difference vegetation index (NDVI), modified normalized difference water index (MNDWI), land use and land cover change (LUCC), population density, water network density, and road network density, with the ecological resistance factors assigned hierarchically according to the natural breakpoint method in ArcGIS 10.8 (Table S1). The ecological corridors were derived by integrating the costly path model with ArcGIS 10.8.

3 $$MCR = f_{min} \mathop \sum \limits_{j = n}^{i = m} \left( {D_{ij} \times R_{i} } \right)$$

$MCR$ refers to the minimum cumulative resistance value from the ecological source site to each of the other points, $f$ refers to an unidentified positive function of the positive connection between the total distance from a location in space to all source landscapes and biological processes, $D_{ij}$ represents the species' spatial distance from landscape unit $i$ to ecological source patch $j$, $R_{i}$ represents the resistance coefficient of the landscape unit $i$ to the movement of certain species, and $min$ is $MCR$ of the target patch to any source landscape.

The gravity model (GM) is a method to determine ecological corridors utilizing interaction intensity between patches that can effectively evaluate the relative importance of potential ecological corridors and how strongly they connect source patches. By using the GM, we can quantify the intensity of interactions between patches and determine which potential corridors are most crucial for maintaining ecological connectivity^40^.

4 $$G = \frac{{M_{i} M_{j} }}{{d^{r} }}$$

The magnitude of the gravitational force between two ecological patches is denoted by $G$. $M_{i} M_{j}$ represents the total ecosystem service value of ecological patches $i$ and $j$ after undergoing standardization and equal weighting. Resistance coefficient ($r$) is defined as the ratio between d and l, where l refers to the minimum distance of the actual biological flow between two ecological patches, and $d$ corresponds to the straight-line distance between two ecological patches)^41^.

#### Ecological network topology algorithm

Complex network theory is utilized in this research to illustrate ecological sources as nodes and ecological pathways as edges of ecological networks. This approach enables the abstraction of landscape space as an undirected network^42,43^, with various topological indicators employed to characterize the ecological network space. In order to evaluate ecological nodes and networks based on their ecological significance, nine topological indicators were chosen (Table S2). The study seeks to identify effective strategies for optimizing the ecological network by establishing correlation between ecological network nodes and carbon sinks.

#### Carbon sink accounting methodology

The total carbon stocks of typical carbon pools, including above-ground, below-ground, soil, and dead organic matter carbon pools, were determined using the carbon sequestration module of the Invest model. Then the following equation computed regional carbon stocks.

5 $$C_{tot} = C_{above} + C_{below} + C_{soil} + C_{dead}$$

The entire carbon stock for this area is $C_{tot}$, The above-ground carbon stock denotes $C_{above}$, $C_{below}$ represents the below-ground root carbon stock, $C_{soil}$ is the soil carbon stock, $C_{dead} { }$ refer to the carbon stock of dead organic matter. For this study, using the average carbon density described in recent literature, we created a database of carbon densities for different land use types in the Beijing–Tianjin–Hebei region (Table S3). We referenced a carbon density database for different land use types in the Beijing–Tianjin–Hebei region by calculating the mean average carbon density reported in four relevant studies^44–47^ conducted in the region.

#### Checking the robustness of complex network connections after network optimization

"Robustness" describes a system's capacity to continue operating well even when changes to its size or structure have been made. Recovery robustness pertains to a network's ability to restore the original properties of its nodes and edges after they have been damaged by natural or human intervention. Node recovered reliability and edge restoration resilience are additional categories for recoverability. On the other hand, connectivity robustness refers to a network's ability to sustain its structural and functional properties, as well as maintain node connectivity and energy transfer, despite external disturbances.

6 $${\text{D}} = 1 - \frac{{N_{r} - N_{d} }}{N}$$

7 $${\text{E}} = 1 - \frac{{M_{r} - {\text{M}}_{e} }}{M}$$

8 $${\text{R}} = \frac{{\text{C}}}{{N - N_{r} }}$$

Node recovery robustness is denoted by D, E refers to the edge recovery robustness, R represents the network connectivity robustness, $N$ refers to the total number of nodes in the network, $N_{r}$ represents the number of nodes that were removed, ${\text{N}}_{d}$ refers to the number of nodes that were recovered after removal, M refers to the total number of edges in the network, $M_{r}$ refers to the number of edges removed from the network, ${\text{M}}_{e}$ represents the number of edges that were recovered after removal. Additionally, ${\text{C}}$ represents the maximum number of nodes in the connected sub-graph of the network after removing certain nodes. By comparing these three measures of robustness before and after network optimization, the effectiveness of ecological spatial network optimization can be validated.

## Analysis of the results

### Building and analyzing ecological spatial networks

#### Ecological source screening

**Figure 2:**
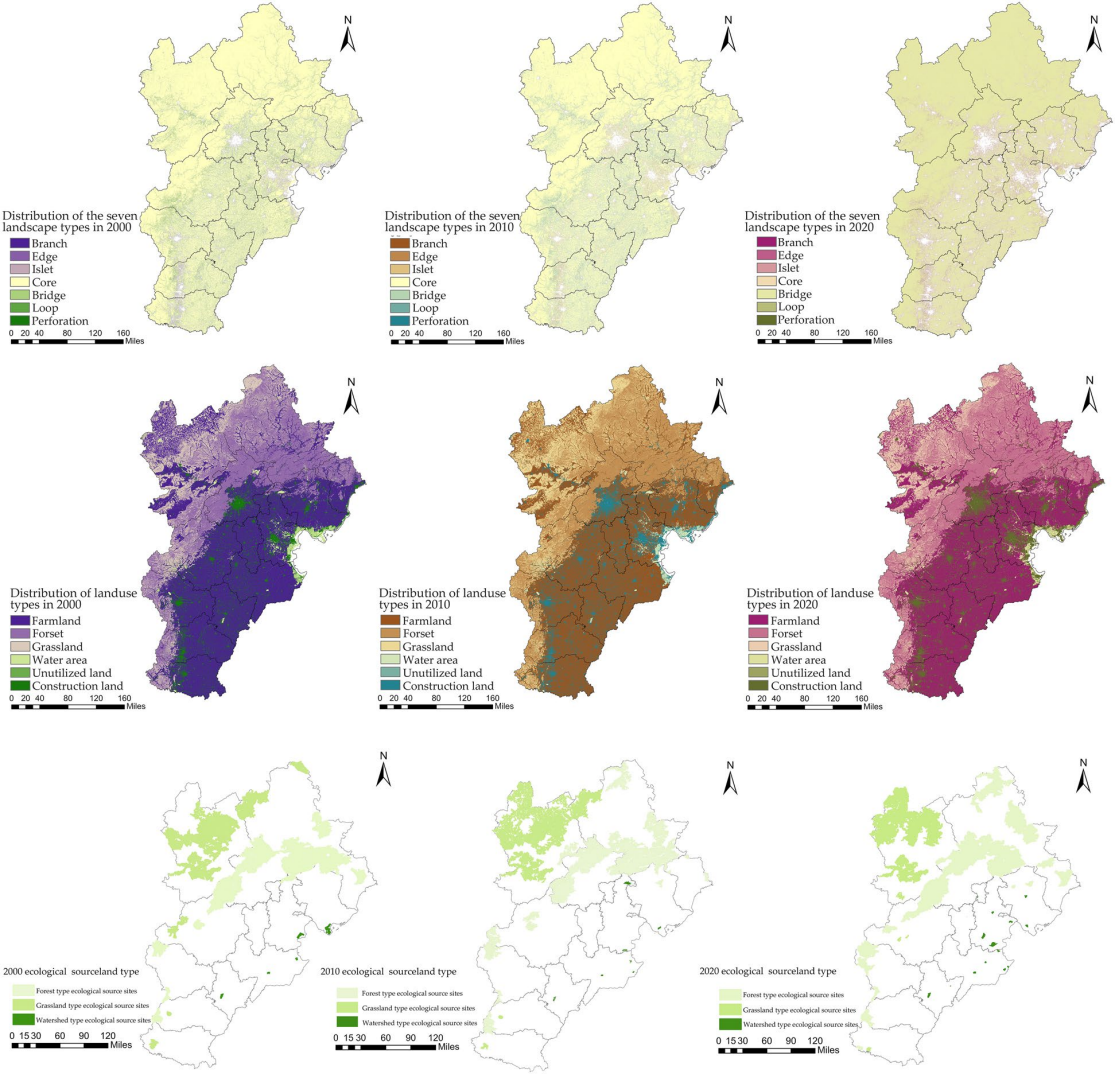
Distribution maps showing seven landscape types identified by MSPA, land use and ecological source types after screening in 2000, 2010 and 2020 (Guidos Toolbox software, ArcGIS 10.8 and Conefor 2.6).

Using the MSPA approach, foreground and background data were imported into GuidosToolbox2.8 software for image analysis in eight areas. The core areas covered 180,629.47 km^2^, 174,388.22 km^2^, and 167,561.90 km^2^, accounting for 45.85%, 44.27%, and 42.54% of the entire landscape area in the research region, respectively. Ecological sources were chosen based on core areas covering more than 25 km^2^, with the landscape interconnection of the three-year core area evaluated utilizing Conefor 2.6 software. The plots' connectivity threshold and interaction likelihood are positioned at 2500 m and 0.5, respectively. Ecological plots with a dPC value > 0.5 were identified, with 217 ecological sources in 2000, 326 ecological sources in 2010 and 412 ecological sources in 2020 identified. Ecological sources of forest, grass, and water, which accounted for the largest area in 13 cities, were finally selected as ecological sources for the subsequent cost path model (Fig. 2). In addition to these ecological sources, weak ecological nodes were also identified by extracting the geographical distribution of ecological source locations in accordance with the idea of extracting “contour lines” in hydrology. These weak ecological nodes were not as significant as the previously identified ecological sources but were still considered in the subsequent cost path model.

#### Investigation of the smallest cumulative ecological resistance surface

By using cost distance analysis to superimpose the resistance values of the eight resistance variables, the geographical distribution of resistance to the ecological security pattern in the Beijing-Tianjin-Hebei study area in 2000, 2010, and 2020 was determined (Fig. S3). The high resistance area corresponds to the distribution of construction land, where the resistance values of flat areas are significantly higher than those of grasslands and forests, with the highest resistance values discovered in and around the urban areas of Beijing, Tianjin, Cangzhou, and Hengshui. This is mainly because these flat areas are predominantly farmland and highly urbanized, while the ecological source areas are small and scattered. The minimum cumulative ecological resistance demonstrated a slight downward trend from 2000 to 2020.

#### Building an ecological spatial network

**Figure 3:**
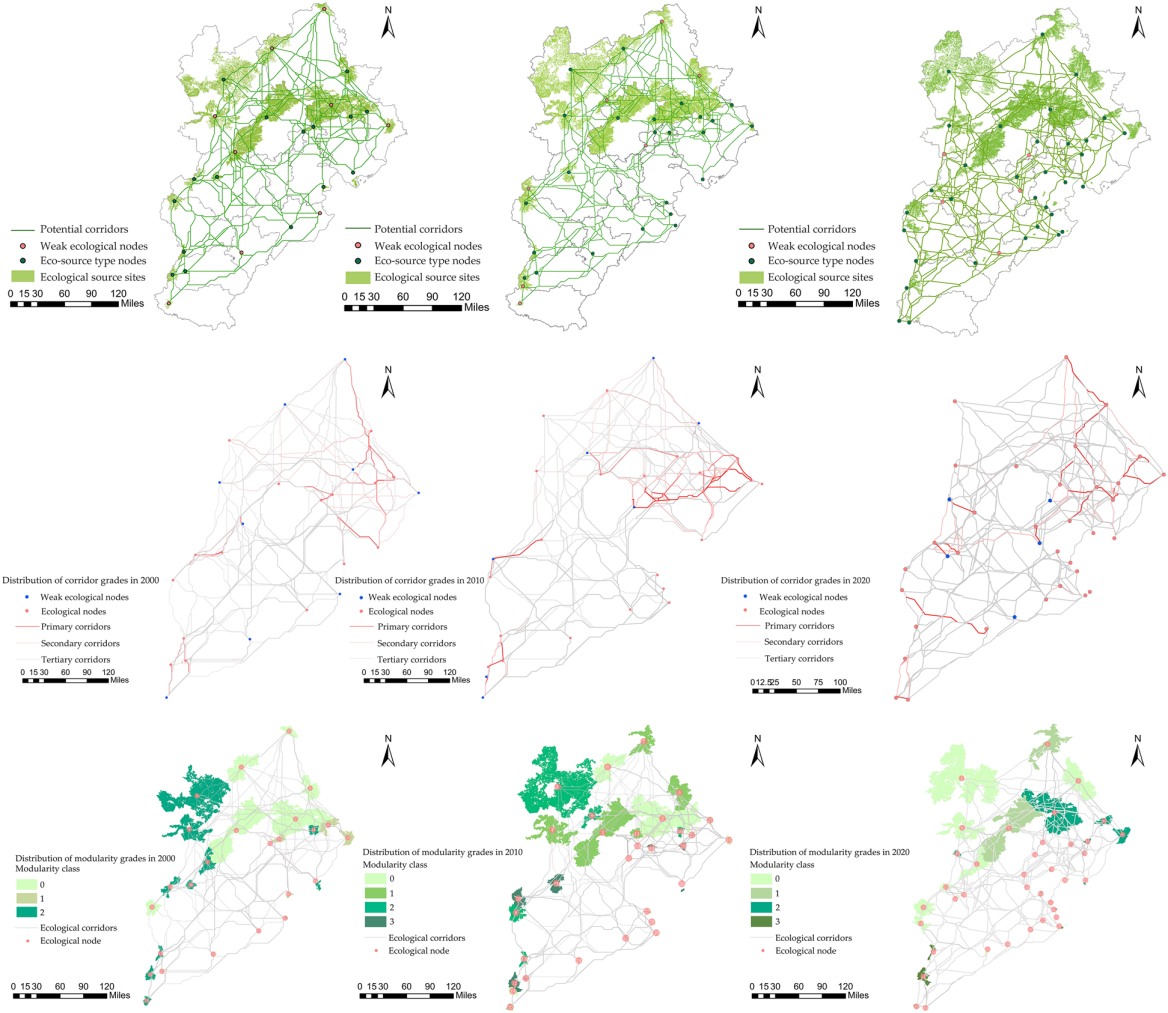
Maps illustrating the distribution of ecological networks, the hierarchical distribution of ecological corridors, and the distribution of the modularization level of ecological source areas in 2000, 2010, and 2020 (ArcGIS 10.8 and Gephi 0.10.1).

Making utilization of the cost path model, potential ecological corridors were generated in 2000, 2010 and 2020, with 296, 462, and 660 corridors, respectively, utilizing the ArcGIS platform's iterator tool (Fig. 3). The northwestern portion of the research area was found to have longer ecological corridors and a denser distribution of ecological source sites than the southeastern portion. The ecological network was observed to be less connected and less stable in the southeastern part. The gravity model was used to create a matrix of connections between ecological source sites in order to evaluate the importance of corridors (Fig. 3). The findings suggest that primary corridors are mostly found near the meeting point of ecological source sites and high-value resistant surfaces, primarily on the eastern side of the research region, and eventually display a decentralized layout. Most frequently seen on the northwestern edge of the research region, secondary corridors extend from the outside to the inside and are centered around the link area between significant ecological source sites. Most often in the southwest of the research region, tertiary corridors are found within small ecological source sites or on the outskirts of urban areas.

The ecological corridors from 2000, 2010, and 2020 were analyzed using the complex network visualization software Gephi, with modularity indices assigned to the ecological source sites to create a distribution of ecological source modularity classes (Fig. 3). Except for 2000, the data show that the ecological spatial network was separated into four communities. This highlights the increasing landscape heterogeneity among ecological sources in 2020 as well as the wider variations between various community networks. Ecological nodes with a variety of modularity is known to be more connected and adaptable in the face of disturbance brought on by humans and naturally aggressive behavior. Therefore, the greater the degree of modularity within a zone, the more stable that area is under disturbance. Conversely, the lower the minimum modularity number, the less secure the area's nodes are less secure and more vulnerable. Additionally, ecological source land type and geospatial location are linked to how community networks are classified, with ecological nodes with the same modularity being more comparable.

The MCR model and community network structure indicate that southeast Beijing, Tianjin, and Hebei have weak ecological underpinnings for ecological sources. It is difficult to move energy and materials around the area, and they have a limited capacity for self-ecological regeneration. They are also less resistant to ecological harm. On the other hand, the central and northern regions benefit from a greater foundation of natural resources, shorter biological corridors, and more effective energy transportation. These areas support ecological self-restoration and are more resistant to external disturbance.

### Network-based analysis

#### Network topology metrics results analysis

**Figure 4:**
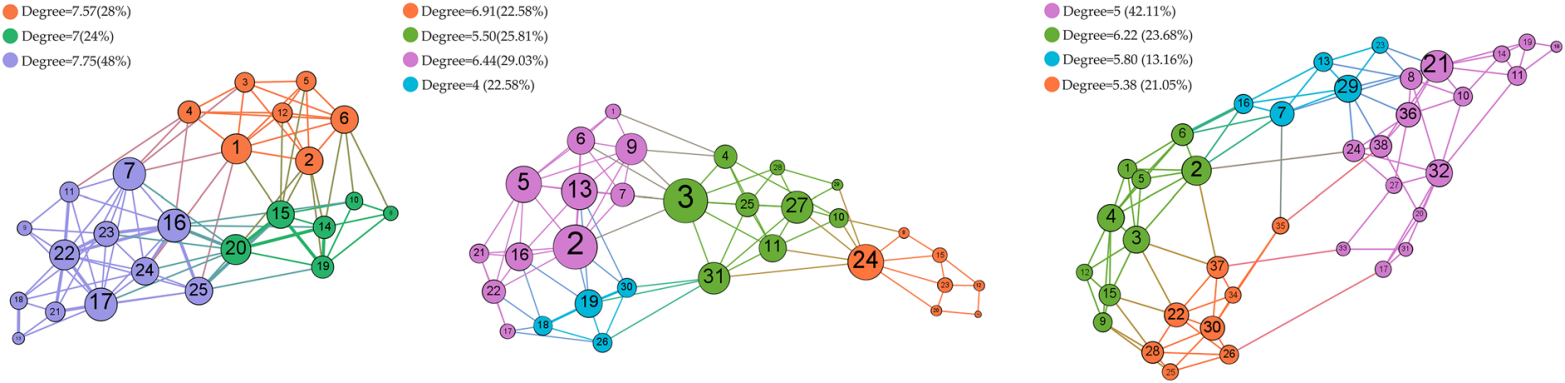
Degree distribution of ecological nodes in 2000, 2010 and 2020 (the size of nodes in the figure corresponds to their degree. with the number on each node indicating its serial number).

As shown in Fig. 4, In 2000, the largest degree nodes 17, 16 and 7 have a value of 11, and are situated in the network's core location with nodes 22, 20 and 1, playing an important connecting role, with the smallest node 13 being 3 and located at the edge of the network. In 2010 the largest degree nodes 2 and 3 had a value of 11, and are situated in the central region of the network with nodes 5, 13 and 24, with the smallest node at the edge of the network. The biggest degree node in the network in 2020 is node 21, which has a degree of 10. Node 18 is the smallest node in the network, with a degree of 2, and is situated at the network's edge. The remaining nodes are spread out around the network and connected to one another to send data.

The ecological spatial network's average topological values from 2000 to 2020 are displayed in Table S4. The degree, clustering coefficient, proximity centrality, and eigenvector centrality of the nodes show a substantial negative trend in the data, indicating a decline in the information flow and the significance of ecological nodes throughout this time. Additionally, the connectivity between nodes decreased, leading to an increase in the average path length. Conversely, the eccentricity and intermediary centrality showed a clear upward trend, indicating that nodes with high eccentricity and intermediary centrality acted a more significant role as "bridges" in the ecological spatial network during this period.

The five topological ecological node indicators were counted (Fig. S4), The study analyzed the overall proximity centrality of the ecological network from 2000 to 2020, finding that it did not change much. In 2000, four nodes had proximity centrality values greater than 0.6, namely nodes 7, 16, 20, and 25, of which nodes 7, 16 and 20 had the highest values at 0.63, indicating their centrality in the network that year. In 2010, nodes 3 and 31 had values greater than 0.5, with node 31 having the highest value of 0.52. In 2020, nodes 2 and 24 had values greater than 0.45, with node 2 having the highest value at 0.47. Additionally, the results present that nodes with high intermediary centrality values were favourable for energy transfer, with node 2 in 2020 having the highest value. In contrast, node 18 in 2020 had zero intermediary centrality, indicating that it was not conducive to the exchange of material and energy. The research analyzed the eigenvector centrality values of the ecological network as well, finding that node 7 in 2000, node 2 in 2010, and node 2 in 2020 had the highest values of 1, indicating their importance in the network. On the other hand, the proximity centrality, intermediary centrality, eigenvector centrality, and PageRank values of node 13 in 2000, node 14 in 2010, and node 18 in 2020 were at their minimum values, suggesting that these nodes were subject to some form of human interference or natural aggression during that period and were not conducive to the exchange of material and energy. Increasing the topological index values of these nodes would enhance the topology of the network. The findings show that the outputs of many topological indicators overlapped, such as nodes 7 and 16 in 2000 and node 2 in 2020 being rated as the most important nodes by several indicators. Therefore, changing these nodes would affect the entire network's topological characteristics.

#### Results of the landscape pattern indicator analysis

FRAGSTATS software was utilized to compute several landscape pattern indices, including patch variety area (CA), landscape shape index (LSI), patch importance index (dPC), maximum patch index (LPI), patch cohesion index (COHESION), landscape fragmentation index (DIVISION), separation (SPLIT), sprawl index (CONTAG), Shannon diversity index (SHDI), and Shannon evenness index (SHEI). These indices were used as important indicators to establish the correlation between ecological source sites and carbon sinks. Landscape pattern indices were obtained for ecological source sites in 2000, 2010, and 2020, respectively (Fig. S4).

The results point to a clear upward trend in the highest values of patch variety area (CA) from 2000 to 2020, with node 1 showing the highest value of ecological source area in 2000 (613,339), 2010 (1,687,600) and 2020 (1,412,400). The trend of change in CA is highly consistent with the maximum patch index (LPI). The overall trends of the patch importance index (dPC), patch cohesion index (COHESION), separateness (SPLIT), and landscape fragmentation index (DIVISION) are stable and do not show significant changes. The maximum patch index (LPI) and landscape shape index (LSI) exhibit a certain downward trend, despite increasing the maximum value of LPI, indicating that the landscape patches are becoming larger. The sprawl index (CONTAG) and Shannon diversity index (SHDI) show a steady increase, with the Shannon evenness index (SHEI) displaying the opposite trend, suggesting a rise in the study area's total landscape heterogeneity and extensibility. However, landscape heterogeneity is still unevenly distributed and locally fragmented.

### Results of carbon stock analysis for nodes, sources and their buffers

Carbon storage averages were calculated for each ecological source site and its surrounding buffer areas for distances of 500 m, 1000 m, 1500 m and 2000 m from 2000 to 2020 (Fig. S5). The results show that, for 12 nodes in 2000, 25 nodes in 2010 and 20 nodes in 2020, the carbon storage averages decreased with an increasing buffer distance. Similarly, for 13 ecological source sites in 2000, 19 ecological source sites in 2010 and 17 ecological source sites in 2020, the carbon storage averages also decreased with an increasing buffer distance. By overlaying the carbon storage values of nodes and source site buffers, we found that nodes 3, 11, and 19 existed in 2000, nodes 5, 7, 8, 10, 16, 19, 22, 25, 26 and 27 existed in 2010 and nodes 21, 25, 27, 28, 30, 31, and 33 showed a decrease in carbon stock values with decreasing buffer distance. However, the carbon stock values of the source sites still kept increasing, indicating that these nodes had a weak influence on carbon storage. It was found that part of the topology index of the nodes themselves was below the average, while the corresponding landscape pattern index values of the source sites were at a high level, thus explaining why the weak influence of the nodes did not affect the carbon storage capacity of the source sites. However, the carbon storage capacity of most of these nodes was significantly lower than that of the nodes and the source sites at the same level. On the other hand, eight nodes in 2000, 15 nodes in 2010 and 14 nodes in 2020 showed a consistent reduction in the average carbon storage value of their source sites, suggesting that enhancing the carbon storage capacity of these nodes could improve the overall average carbon storage capacity of the source sites. Therefore, these nodes are the primary targets for optimization in the subsequent network optimization of this study.

## Discussion

### Relevance analysis

#### Relevance of important ecological nodes to carbon sinks

**Figure 5:**
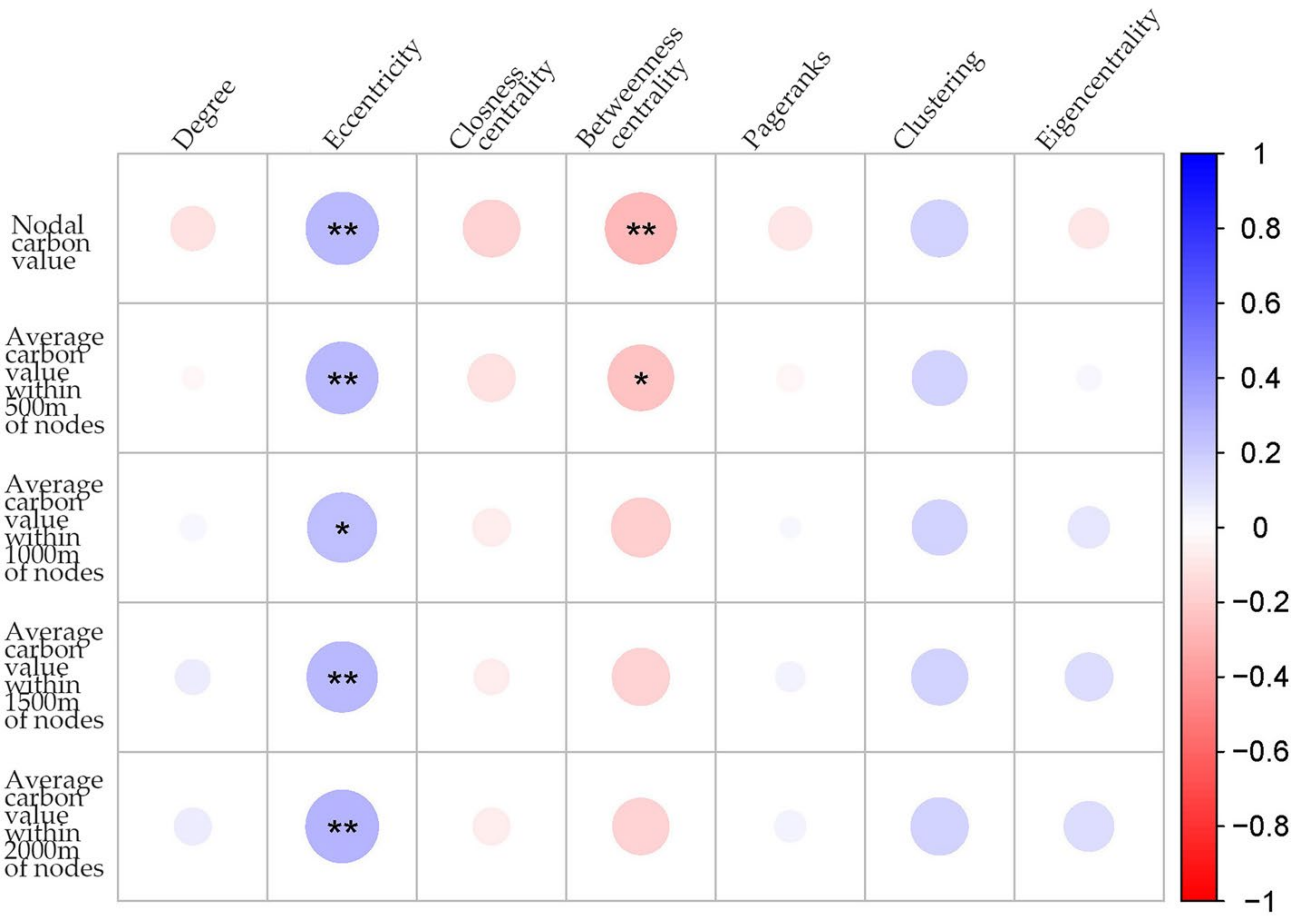
Pearson correlation coefficient between ecological node topological indicators and carbon sinks (asterisk indicates statistical significance: 0.01 ≤ *P < 0.05, 0.001 ≤ **P < 0.01, ***P < 0.001).

We imported the carbon sink values of each ecological node and the average carbon sink values within buffer zones at various distances (500 m, 1000 m, 1500 m, and 2000 m) into R-studio with the aim of investigating the relationship between topological markers of ecological nodes and their capacity to sequester carbon. We then conducted the Pearson correlation analysis, with the results presented in Fig. 5. The analysis showed that eccentricity positively correlated with carbon sink values, whereas intermediate centrality negatively correlated with carbon sink values. Interestingly, the negative correlation between intermediate centrality and carbon sinks weakened as the distance from the node increased.

#### Relevance of ecologically important source sites to carbon sinks

**Figure 6:**
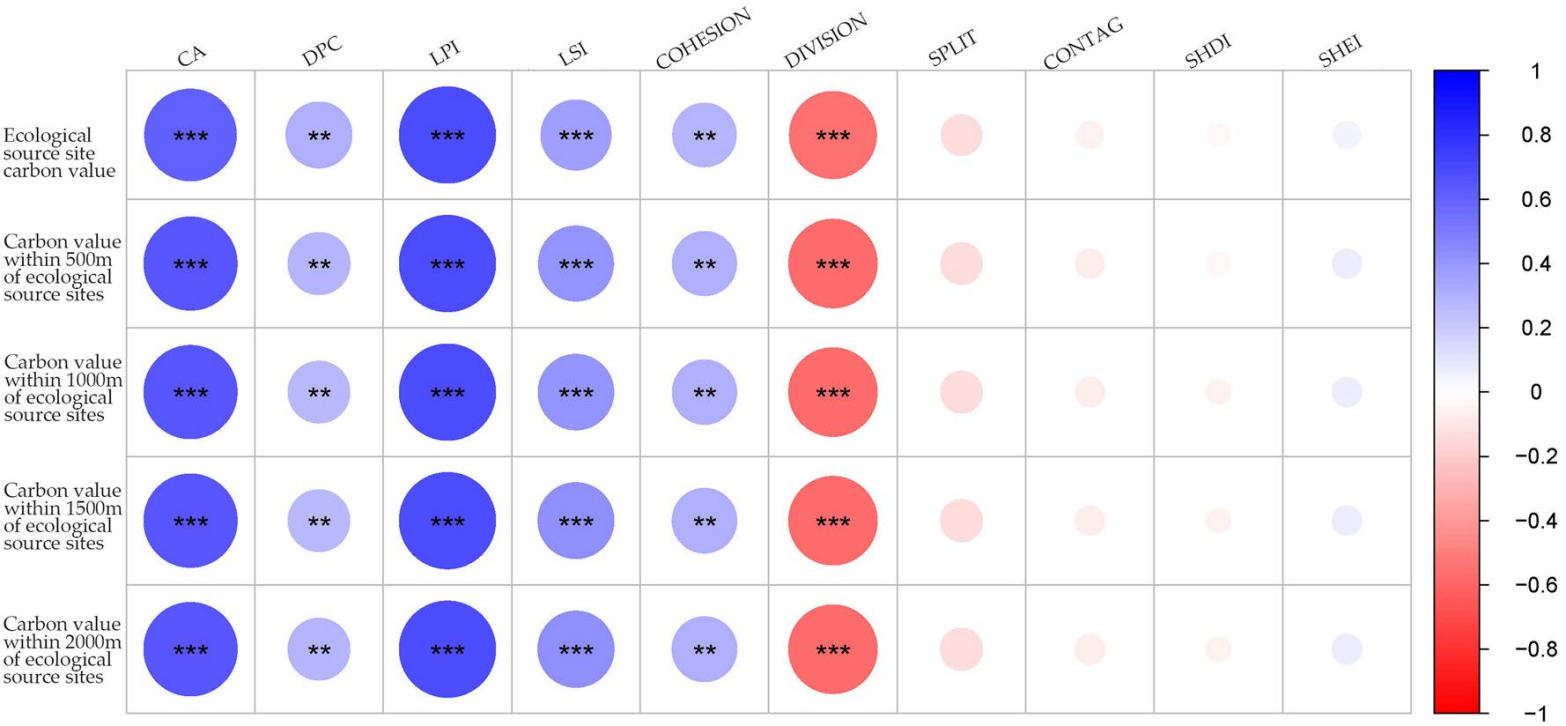
Pearson correlation coefficient between landscape pattern index of ecological source and carbon sinks (asterisk indicates statistical significance: 0.01 ≤ *P < 0.05, 0.001 ≤ **P < 0.01, ***P < 0.001).

The Pearson correlation analysis was employed to examine the relationship between the landscape pattern indicators of ecological source sites and the carbon sequestration capacity of the ecosystems in which they are located. The carbon sink values were calculated for each ecological source site within 500 m, 1000 m, 1500 m and 2000 m from the source site (Fig. 6). The results indicated that several indicators, including patch cohesion index (COHESION), maximum patch index (LPI), patch variety area (CA), patch importance index (dPC) and landscape shape index (LSI) significantly positively correlated with the carbon sinks of the ecological source sites. Conversely, the landscape fragmentation index (DIVISION) indicated a substantial inverse relationship with the carbon sinks of ecological source sites.

#### Correlation between ecological nodes and ecological nodes and ecological source model indices

**Figure 7:**
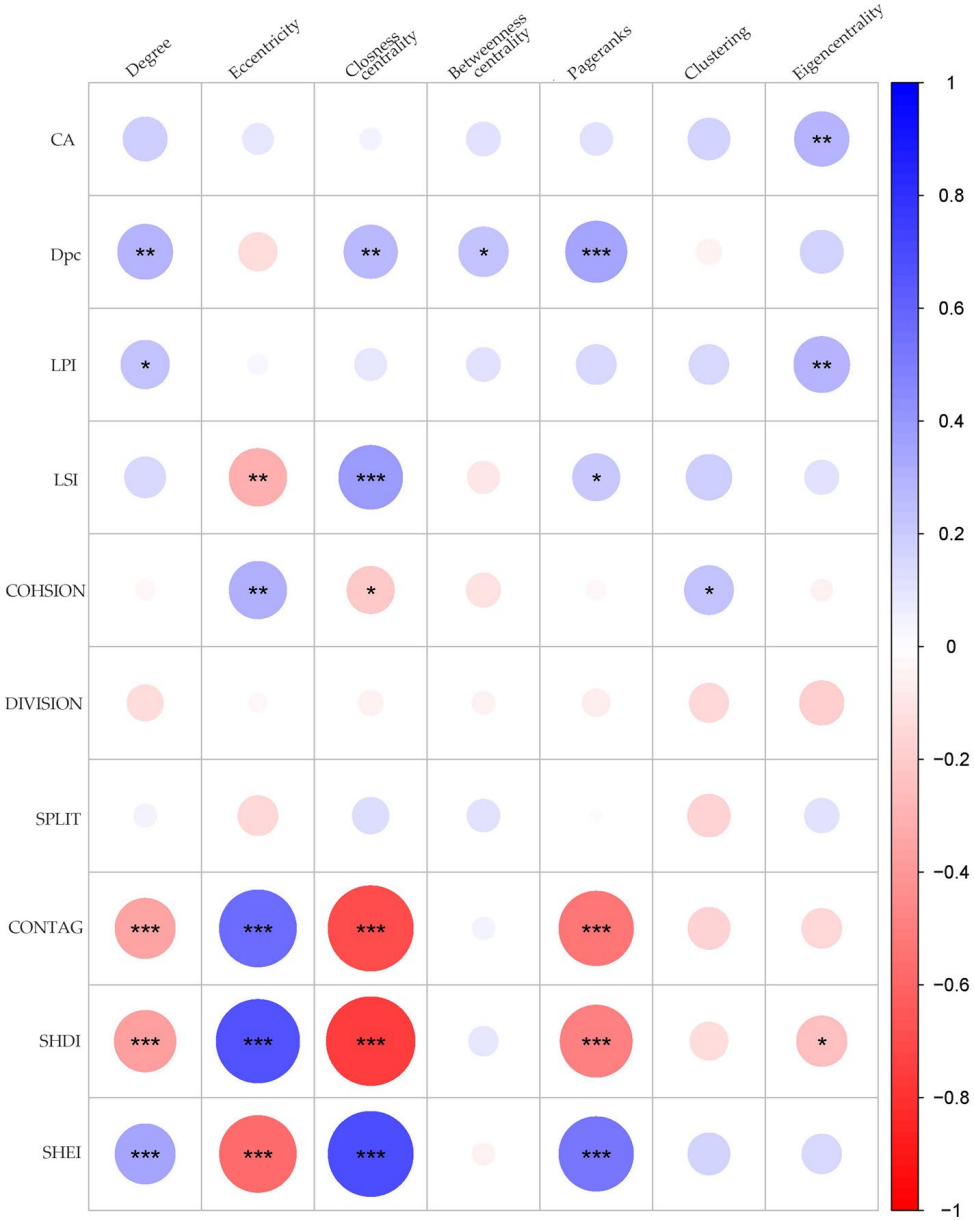
The correlation coefficient between the landscape pattern index and topological index of ecological nodes in the ecological source area (asterisk indicates statistical significance: 0.01 ≤ *P < 0.05, 0.001 ≤ **P < 0.01, ***P < 0.001).

Upon examining Figs. 5, 6, and 7, there is a positive correlation between eccentricity and COHESION within the group, as well as a positive correlation between eccentricity and carbon sinks within the node and source buffer. These results suggest that improving the eccentricity and COHESION of topological nodes can increase the contribution of these nodes to enhance the carbon storage benefits in the source areas.

### Optimization recommendations

In order to increase carbon values in source areas, we aimed to homogeneously reduce carbon values in nodes and source areas. We have identified weak carbon sink nodes that consistently showed a reduction in the average carbon storage value of both nodes and source areas from 2000 to 2020, based on the absolute value of carbon difference between nodes and source areas. We then adjusted the network topology by adding stepping stones and ecological corridors to optimize the network, while determining the order and levels of optimizable nodes to improve the carbon storage capacity of nodes and source areas. For instance, we improved the eccentricity by increasing ecological corridors between adjacent nodes and improved the cohesiveness index (COHESION) of the patches by adding ecological stepping stones. The final results included 25 nodes in the forest category, ranked in descending order of absolute value as 22 in 2020, 30 in 2010, 18 in 2010, 14 in 2000 and 18 in 2020; 5 nodes in the grassland category, including 25 in 2000; and 7 nodes in the watershed category, including 35 in 2020 and 17 in 2000 (Fig. S6). Ultimately, in 2000, 11 ecological stepping stones and 36 ecological corridors were added. In 2010 there were 5 ecological stepping stones and 10 ecological corridors, and in 2020 there will be 11 ecological stepping stones and 55 ecological corridors. The length of ecological corridors was successfully decreased, and the nodes of the optimized eco-spatial network were more closely connected to one another, facilitating the flow of energy and the exchange of tangible data even more.

### Robustness comparison before and after optimization

**Figure 8:**
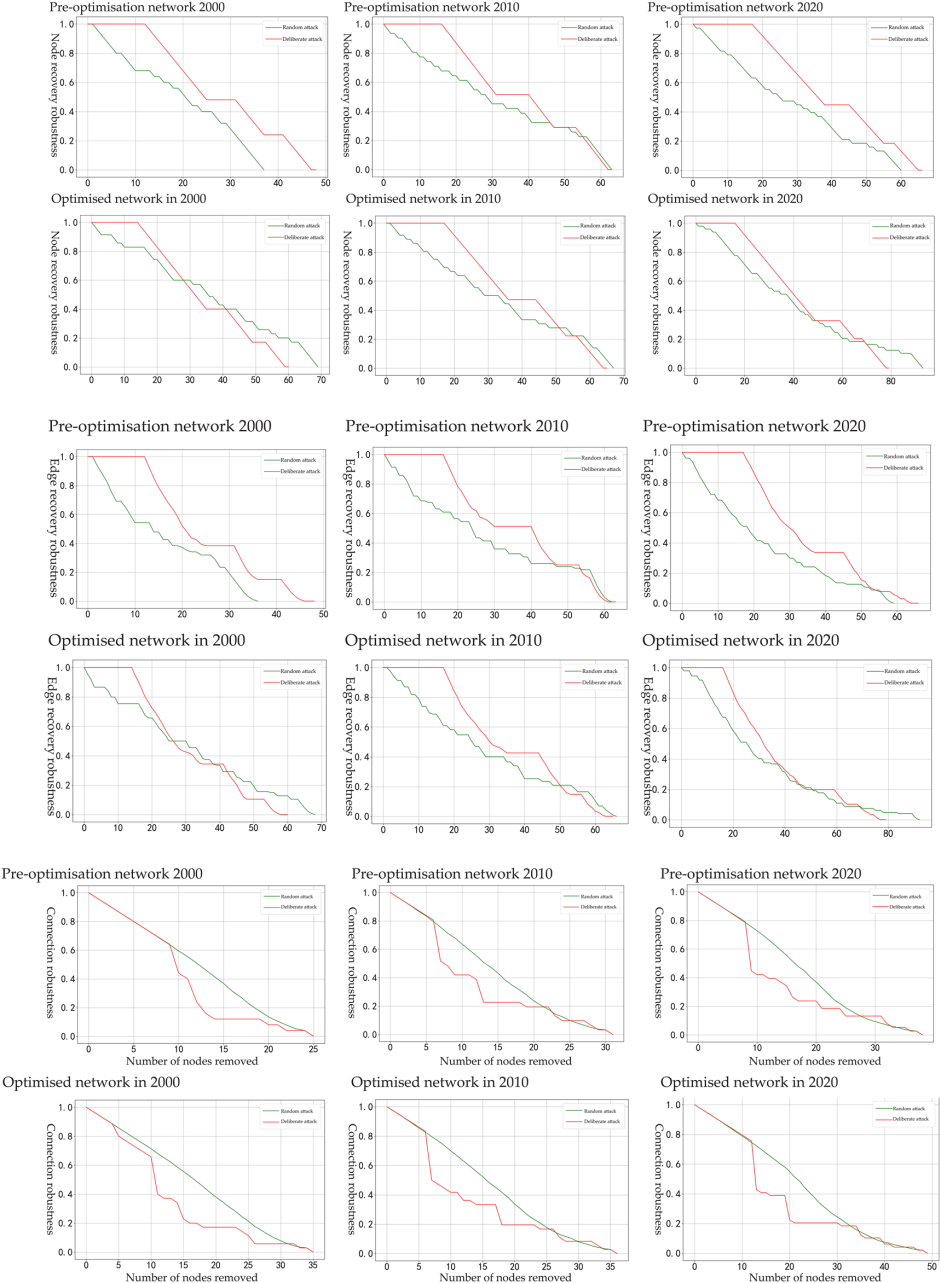
Robustness test of network nodes, edges and connectivity before and after optimization in the Beijing-Tianjin-Hebei research area in 2000, 2010, and 2020.

In ecological cyber-space simulations, arbitrary and malicious assaults on the network can be utilized to gauge the severity of ecological harm that the landscape space has endured. The change in network robustness can be measured to evaluate the network's resistance to external damage before and after the optimization of the cyberspace simulation for the Beijing–Tianjin–Hebei study area. The findings indicate that node recovery resilience, edge recovery robustness, and connection robustness (Fig. 8) are all somewhat improved, with the network connection robustness experiencing the greatest improvement. As the proportion of nodes vulnerable to arbitrary or malicious attacks grew, the network's robustness decreased before and after optimization. However, when submitted to random assaults, the network's stability was substantially higher than when subjected to intentional attacks. There were also differences in the changes in robustness among the various robustness kinds. Combining the performance of the three robustness types, we can conclude an improvement in the stability and ecological resilience of the optimized ecological space network.

The recovery robustness scenario of nodes reveals that the optimized network exhibits a more pronounced convex curve compared to the pre-optimized network curve, indicating that the optimized network has a slower rate of decline and a delayed trend in decline's inflection point. When subjected to malicious attacks, the robustness of the optimized network begins to decline below 1 at the 15th node and below 0.1 at the 55th node in 2000, eventually leading to network collapse. In 2010, the robustness started to fall below 1 at the 18th node and below 0.1 at the 65th node, leading to network collapse. In 2020, the robustness began to decrease below 1 at the 18th node and below 0.1 at the 66th node, leading to network collapse. On the other hand, after random and malicious attacks, the robustness of the optimized network reached the last node and remained at 0.4 and 0.5 in 2000, 0.5 and 0.48 in 2010 and 0.3 and 0.2 in 2020, respectively. These values are all superior to the robustness of the network prior to optimization.

In the scenario of edge restoration robustness, the network demonstrated a partial stability over three years when subjected to malicious attacks. However, it showed a relatively stable rate of decline under random attacks. The optimized network displayed greater stability compared to the pre-optimized network. This was evident from the significantly slower decline rate in the robustness of the optimized network when unintentionally harmed, as well as the reduced number of nodes with robustness below 0.1.

The stability of the optimized network significantly improved from 2000 to 2020 under both malicious and random attacks, with the network connectivity robustness decreasing at a significantly slower rate compared to the pre-optimized network. In 2000, the pre-optimized network's robustness fell below 0.1 when node 20 was maliciously attacked, causing the network to collapse, while the optimized network's robustness did not fall below 0.1 until node 26 was attacked. In 2010, the pre-optimized network maintained 0.2 connectivity with 18–19 nodes removed, while the post-optimized network maintained 0.2 connectivity with only 16–17 nodes removed. In 2020, the effect of optimizing the network was more evident after node 20, where the pre-optimized network maintained 0.12 connectivity with 24–32 nodes removed, while the post-optimized network maintained 0.12 connectivity. Furthermore, after node 20, the post-optimized network maintained 0.2 connectivity after removing 20–30 nodes, whereas the pre-optimized network only maintained 0.12 connectivity after removing 24–32 nodes.

### Comparison of carbon sink stocks before and after optimization

In this research, before and after optimizing the ecological network in the Beijing-Tianjin-Hebei region, we applied the Invest model carbon storage module measurement method to calculate the carbon sink values using Eq. (5) (Table S3). Our findings indicate that the ecological source carbon storage increased by 28,141,989.1 tons, and the ecological corridor carbon storage increased by 4018.23 tons in 2000. In 2010, the ecological source carbon storage increased by 2,355,805.9 tons and the ecological corridor carbon storage increased by 759.6 tons. By 2020, the carbon stock of ecological sources is projected to increase by 19,962,554.46 tones. The carbon stock of ecological corridors is projected to increase by 2453.04 tons, representing a significant improvement (Table S5).

## Conclusions

Compared with the results of other similar studies, the topological indices of carbon sinks are inconsistent due to the different carbon accounting methods, spatial objects and research objectives. Still, the intermediary centrality topological index clearly shows a positive and negative correlation with various carbon sink measurement methods, which is sufficient to prove the importance of future research on this topological index in the study of sink enhancement networks. The research institute proposed a framework for increasing the synergistic accumulation of urban green network nodes and source areas, which can be applied to various urban spaces at multiple scales. Due to the close relationship between carbon capacity and climate, the strongly correlated network structural factors in different regions may vary. However, this does not hinder the adjustment of the overall network layout based on the strongly correlated factors identified by the framework, thereby promoting the maximum synergistic accumulation effect of the network.

In conclusion, we constructed the ecological network space for the Beijing-Tianjin-Hebei study area in 2000, 2010 and 2020, and calculated various network topological indicators for the network nodes and landscape pattern indices for the corresponding source areas. Using the Invest model, we also estimated the carbon storage capacity of ecological nodes, source regions, and their buffer zones. The relationship between node topological indicators, landscape pattern indices, and carbon sequestration capacity was then analyzed, and recommendations were made to optimize the network in a carbon–neutral context. Our primary findings are the following:

1. In 2000, 2010 and 2020, the spatial clustering coefficients of the ecological network in Beijing, Tianjin, and Hebei were large, indicating small-world characteristics and an overall structure that is relatively stable.
2. By enhancing the eccentricity of the node topology, the patch cohesion index (COHESION) of the ecological source site can maximize the contribution of the node to enhance the carbon stock benefits of the source site.
3. We propose an edge-increasing strategy to optimize the ecological spatial networks in Beijing, Tianjin, and Hebei in order to enhance the carbon sequestration capacity of various ecological elements. Additionally, we tested the state before and after network optimization for robustness to ensure the effectiveness of the edge-increasing strategy.

While this study has contributed to the field, some limitations still need to be addressed. The ecological spatial network concept is still in its theoretical stage, with further empirical research required to estimate the effectiveness of optimization strategies. Additionally, the construction of ecological corridors incurs significant economic costs, making it essential to weigh these costs against the potential ecological benefits carefully. Further research is needed to provide a more comprehensive assessment of the economic and ecological feasibility of such projects.

### Supplementary Information

Supplementary Information.

## Data Availability

All data generated or analyzed during this study are included in this published article and the supplementary information file.
