# Supplementary material for: An empirical assessment of whether urban green ecological networks have the capacity to store higher levels of carbon
Source: Sci Rep. 2024 Feb 7;14:3132. doi: 10.1038/s41598-024-52650-y (PMC10850523; doi:10.1038/s41598-024-52650-y)
Supplement: Supplementary file 1 — Supplementary Information. [file 41598_2024_52650_MOESM1_ESM.docx]

# Appendix S for Supplemental Materials

| **Factor** | **Value** | **Weight** | **Grade** | **Factor** | **Value** | **Weight** | **Grade** |
| --- | --- | --- | --- | --- | --- | --- | --- |
| DEM/m | -57 — 231 | 0.154 | 1 | LUCC | Forest, Water, Wetland | 0.144 | 1 |
|  | 231 — 590 |  | 3 |  | Grassland |  | 3 |
|  | 590 — 935 |  | 5 |  | Farmland |  | 5 |
|  | 935 — 1299 |  | 7 |  | Construction land |  | 7 |
|  | 1299 — 2849 |  | 9 |  | Utilized land |  | 9 |
| SLOPE/(◦) | 0 — 5.21 | 0.147 | 1 | Population density | 0 — 27.49 | 0.136 | 1 |
|  | 5.21 — 12.58 |  | 3 |  | 27.49 — 119.13 |  | 3 |
|  | 12.58 — 20.25 |  | 5 |  | 119.13 — 293.25 |  | 5 |
|  | 20.25 — 29.45 |  | 7 |  | 293.25 — 659.82 |  | 7 |
|  | 29.45 — 78.22 |  | 9 |  | 659.82 — 2346.02 |  | 9 |
| MNDWI | -0.86 — -0.46 | 0.104 | 9 | NDVI | 0 — 0.3 | 0.108 | 9 |
|  | -0.46 — -0.33 |  | 7 |  | 0.3 — 0.44 |  | 7 |
|  | -0.33 — -0.11 |  | 5 |  | 0.44 — 0.58 |  | 5 |
|  | -0.11 — 0.32 |  | 3 |  | 0.58 — 0. 75 |  | 3 |
|  | 0.32 — 0.93 |  | 1 |  | 0.75 — 0.92 |  | 1 |
| Water network density | 0 — 6.82 | 0.103 | 9 | Road network density | 0 —12.95 | 0.104 | 1 |
|  | 6.82 — 13.58 |  | 7 |  | 12.95 — 40.21 |  | 3 |
|  | 13.58 — 22.22 |  | 5 |  | 40.21 — 93.76 |  | 5 |
|  | 22.22 — 35.12 |  | 3 |  | 93.76 — 198.75 |  | 7 |
|  | 35.12 — 54.89 |  | 1 |  | 198.75 — 389.18 |  | 9 |

**Table S1.** Resistance factors and their weights

| **Index type** | **Name of index** | **Introduction of algorithm** | **Significance of indicators** | **References** |
| --- | --- | --- | --- | --- |
| Node assessment | Degree | The quantity of ecological corridors that an ecological node has | Describe the number of other nodes connected to this node | [1, 2] |
|  | Eccentricity | Indicates the corridor distance from a given starting point to the node furthest away from it | Farthest shortest path distance from a node to other nodes | [3] |
|  | clustering | The proportion of corridors really linking a node's surrounding nodes to all other feasible corridors | Indicates the extent to which a node domain is linked to each other | [4, 5] |
|  | Closeness centrality | The reciprocal of the product of the shortest paths between all nodes multiplied by the quantity of nodes | Denotes the distance of the ecological node from other nodes via the shortest path; also quantifies the network geometric centre of the ecological node | [6] |
|  | Betweenness centrality | The percentage of all shortest paths in a complex network that pass by a node. | denotes the proportion of the shortest path of an ecological node to any two nodes in the network | [1] |
|  | Eigenvector centrality | A relative score is given for every eco site. | Used to assess the importance of nodes | [7] |
|  | Average path length | The network's average of the least distance between any two ecological nodes | Indicates the fluidity of energy flow across the ecological spatial network | [3, 8] |
| Network evaluation | Clustering coefficient | Average of every node in the network's clustering coefficients | Indicates whether  the network's ecological nodes are likely to be distributed centrally or decentralized. | [2, 8] |
|  | Modularity | An indicator of the network's community structure's efficiency, with each ecological node in the network being assigned to a different community | Evaluating the effectiveness of online community segmentation | [8, 9] |

**Table S2.** Topology indicators

| **Code** | **Land use** | **C_Above_** | **C_Below_** | **C_Soil_** | **C_Dead_** |
| --- | --- | --- | --- | --- | --- |
| 1 | Farmland | 8.88 | 32.14 | 86.8 | 6.55 |
| 2 | Forests | 28.38 | 47.77 | 152.37 | 10.41 |
| 3 | Grassland | 17.6 | 35.79 | 82.11 | 7.15 |
| 4 | Water area | 3.98 | 18.43 | 3.85 | 0 |
| 5 | Unutilized land | 5.44 | 15.53 | 33.7 | 0 |
| 6 | Construction land | 2.94 | 10.7 | 59.33 | 0 |
| 7 | Wetlands | 4.85 | 12.15 | 132.48 | 0.62 |

**Table S3.** Carbon storage values of urban land cover types

| **Indicators/Years** | **2000** | **2010** | **2020** |
| --- | --- | --- | --- |
| Degree | 7.5200 | 5.9355 | 5.4737 |
| Eccentricity | 3.2000 | 4.6452 | 4.6579 |
| clustering | 0.5528 | 0.5289 | 0.4807 |
| Closeness centrality | 0.5300 | 0.3965 | 0.3699 |
| Betweenness centrality | 0.0400 | 0.0550 | 0.0484 |
| Eigenvector centrality | 0.6317 | 0.4479 | 0.5188 |
| Average path length | 1.9200 | 2.5960 | 2.7410 |
| Clustering coefficient | 0.5530 | 0.5290 | 0.4810 |

**Table S4.** The ecological spatial network's average topological values in 2000, 2010 and 2020

| **Type** | **Carbon sink of source (ton)** | **Carbon sink of the corridor (ton)** |
| --- | --- | --- |
| Not optimized in 2000 | 547928802.60 | 72954.23 |
| Optimization 2000 | 576070788.70 | 76972.46 |
| Not optimized in 2010 | 682506102.00 | 77363.07 |
| Optimization 2010 | 684861907.9 | 78122.73 |
| Not optimized in 2020 | 652375174.10 | 82415.14 |
| Optimization 2020 | 672336968.90 | 84868.18 |

**Table S5.** Carbon sink changes in 2000, 2010, and 2020 before and after ecological spatial network optimization


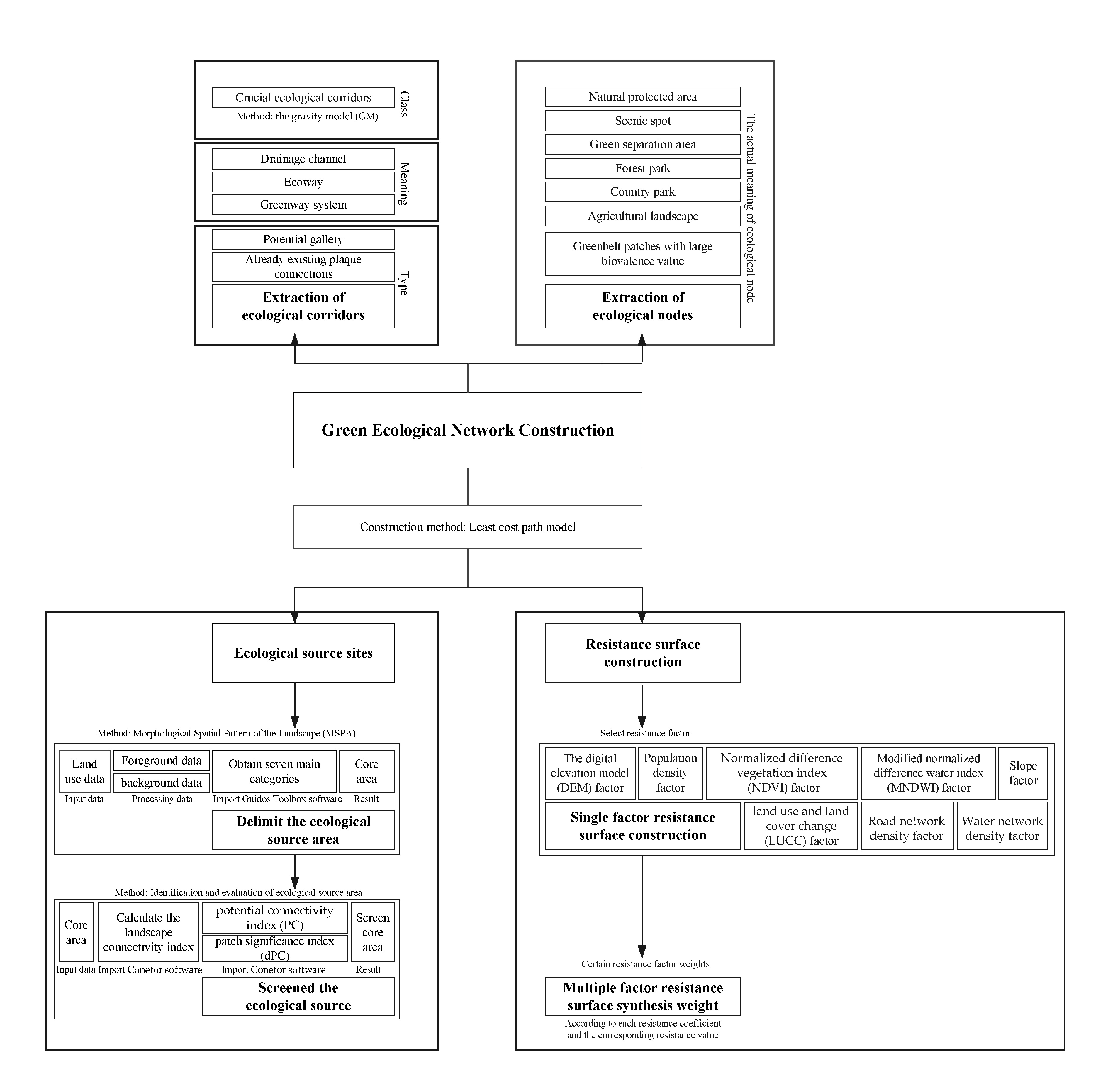


**Figure S1.** Urban green ecological networks model illustration





**Figure S2.** The dataset for the Beijing-Tianjin-Hebei area, visualized using ArcGIS 10.8


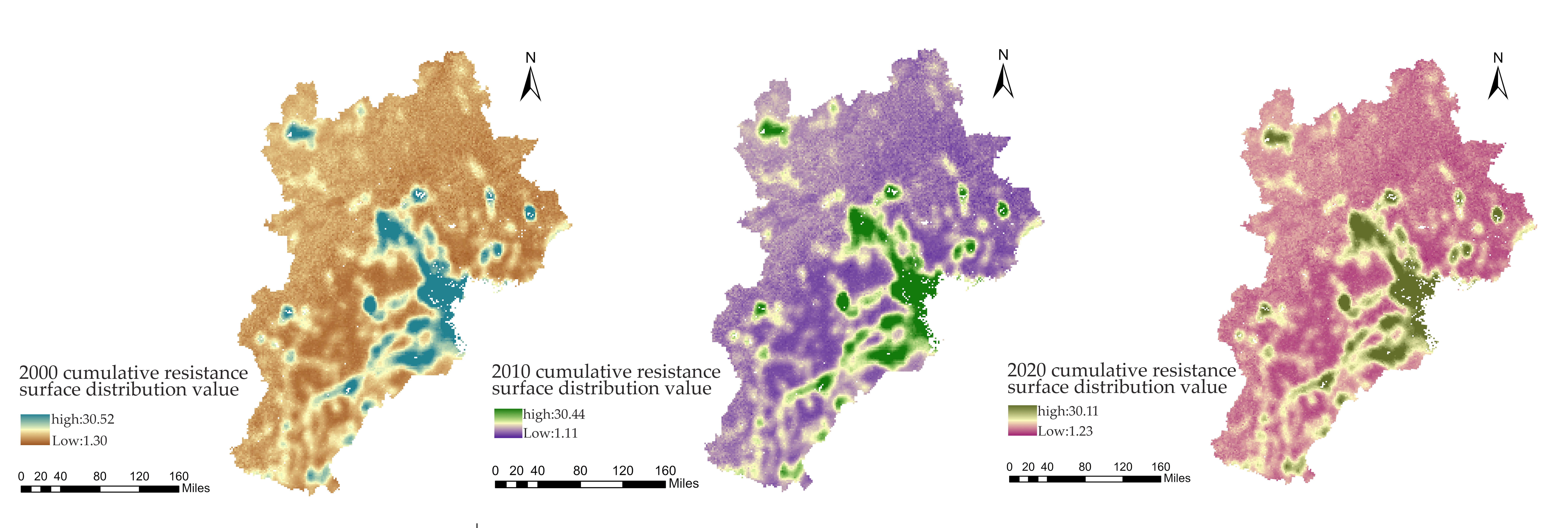


**Figure S3.** Surface distribution of cumulative resistance in 2000, 2010, and 2020 (ArcGIS 10.8)





**Figure S4.** Results of six ecological node topological indicators and landscape pattern index of the ecological source area in 2000, 2010 and 2020


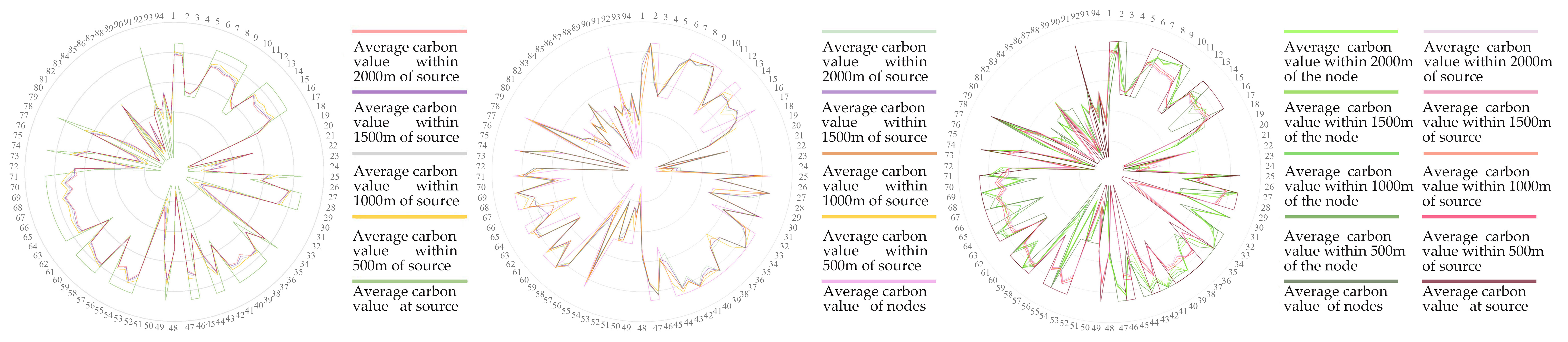


**Figure S5.** Changes of carbon storage values at different distance nodes and sources in 2000, 2010 and 2020

*
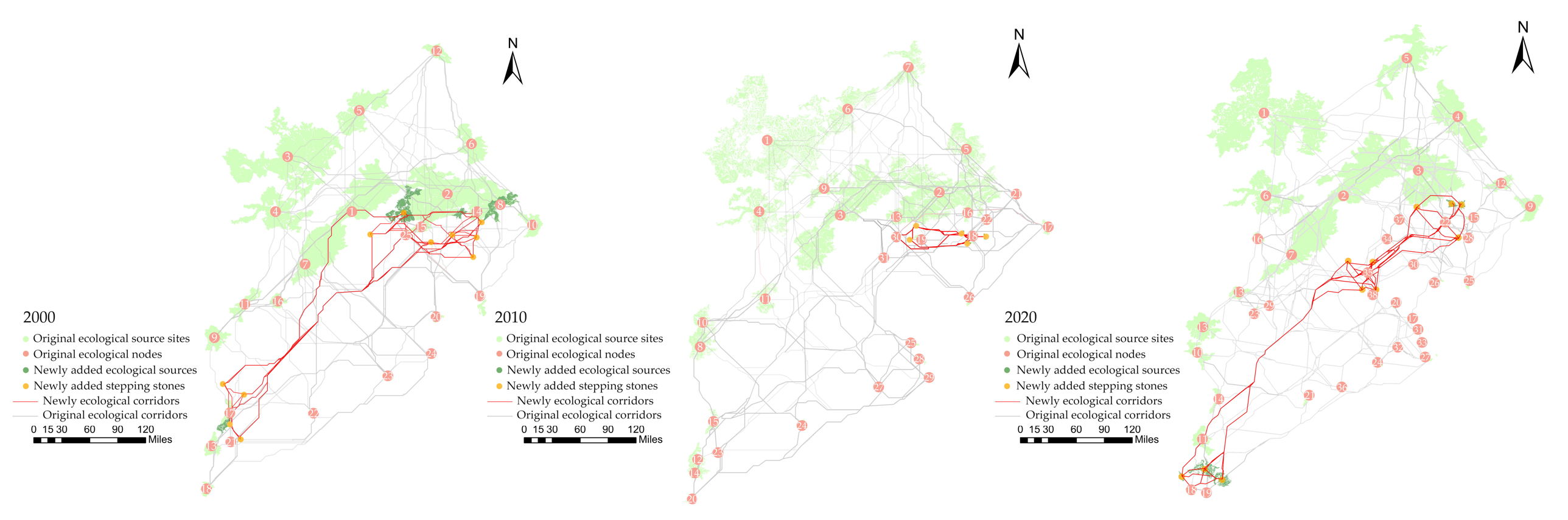
*

**Figure S6.** The optimal ecological spatial network of the Beijing-Tianjin-Hebei research area in the years 2000, 2010, and 2020 (ArcGIS10.8)

**References**

Cárdenas, J. P., Santiago, A., Tarquis, A. M., Losada, J. C., Borondo, F., Benito, R. M. Soil porous system as heterogeneous complex network. *GEODERMA.* **160**, 13-21 (2010).

Costa, L. D. F., Rodrigues, F. A., Travieso, G., Villas Boas, P. R. Characterization of complex networks: A survey of measurements. *ADV PHYS.* ***56***, 167-242 (2007).

Brandes, U. A faster algorithm for betweenness centrality. *J MATH SOCIOL*. ***25***, 163-177 (2001).

Chen, L., Lu, J. A. Cluster synchronization in a complex dynamical network with two nonidentical clusters. *Journal of Systems Science and Complexity*. **21**, 20-33 (2008).

Lancichinetti, A., Fortunato, S. Consensus clustering in complex networks. *SCI REP-U.* **2**, 1-7 (2012).

Koschützki, D., Lehmann, K. A., Peeters, L., Richter, S., Tenfelde-Podehl, D., Zlotowski, O. Centrality indices. *Network analysis: methodological foundations.* 16-61 (2005).

Qiu, S., Yu, Q., Niu, T.,, Fang, M., Guo, H., Liu, H., Li, S. Study on the Landscape Space of Typical Mining Areas in Xuzhou City from 2000 to 2020 and Optimization Strategies for Carbon Sink Enhancement. *REMOTE SENS-BASEL*. **14**, 4185 (2022).

Latapy, M. Main-memory triangle computations for very large (sparse (power-law)) graphs. *THEOR COMPUT SCI*. **407**, 458-473 (2008).

Newman, M. E. Modularity and community structure in networks. *Proceedings of the national academy of sciences*. ***103***, 8577-8582 (2006).
